# Pooled-DNA sequencing identifies genomic regions of selection in Nigerian isolates *of Plasmodium falciparum*

**DOI:** 10.1186/s13071-017-2260-z

**Published:** 2017-06-29

**Authors:** Kolapo M. Oyebola, Emmanuel T. Idowu, Yetunde A. Olukosi, Taiwo S. Awolola, Alfred Amambua-Ngwa

**Affiliations:** 1 Medical Research Council Unit The Gambia, Atlantic Road, Fajara, Gambia; 2 Parasitology and Bioinformatics, Department of Zoology, Faculty of Science, University of Lagos, Lagos, Nigeria; 3 Nigerian Institute of Medical Research, Lagos, Nigeria

## Abstract

**Background:**

The burden of falciparum malaria is especially high in sub-Saharan Africa. Differences in pressure from host immunity and antimalarial drugs lead to adaptive changes responsible for high level of genetic variations within and between the parasite populations. Population-specific genetic studies to survey for genes under positive or balancing selection resulting from drug pressure or host immunity will allow for refinement of interventions.

**Methods:**

We performed a pooled sequencing (pool-seq) of the genomes of 100 *Plasmodium falciparum* isolates from Nigeria. We explored allele-frequency based neutrality test (Tajima’s D) and integrated haplotype score (iHS) to identify genes under selection.

**Results:**

Fourteen shared iHS regions that had at least 2 SNPs with a score > 2.5 were identified. These regions code for genes that were likely to have been under strong directional selection. Two of these genes were the chloroquine resistance transporter (CRT) on chromosome 7 and the multidrug resistance 1 (MDR1) on chromosome 5. There was a weak signature of selection in the dihydrofolate reductase (DHFR) gene on chromosome 4 and MDR5 genes on chromosome 13, with only 2 and 3 SNPs respectively identified within the iHS window. We observed strong selection pressure attributable to continued chloroquine and sulfadoxine-pyrimethamine use despite their official proscription for the treatment of uncomplicated malaria. There was also a major selective sweep on chromosome 6 which had 32 SNPs within the shared iHS region. Tajima’s D of circumsporozoite protein (CSP), erythrocyte-binding antigen (EBA-175), merozoite surface proteins - MSP3 and MSP7, merozoite surface protein duffy binding-like (MSPDBL2) and serine repeat antigen (SERA-5) were 1.38, 1.29, 0.73, 0.84 and 0.21, respectively.

**Conclusion:**

We have demonstrated the use of pool-seq to understand genomic patterns of selection and variability in *P. falciparum* from Nigeria, which bears the highest burden of infections. This investigation identified known genomic signatures of selection from drug pressure and host immunity. This is evidence that *P. falciparum* populations explore common adaptive strategies that can be targeted for the development of new interventions.

**Electronic supplementary material:**

The online version of this article (doi:10.1186/s13071-017-2260-z) contains supplementary material, which is available to authorized users.

## Background

Malaria is a parasitic disease responsible for morbidity in approximately 214 million people globally and an estimated 438,000 deaths [1]. In the last two decades, there have been significant decline in malaria prevalence due to interventions with drug, long-lasting insecticidal nets and other vectorial interventions. However, the disease remains a significant public health problem in sub-Saharan Africa (sSA) which harbours over 90% of the worldwide burden [1]. The endemicity in sub-Saharan Africa is heterogeneous, with some regions heading towards elimination phase while hotspots of transmission remain in others. This heterogeneity is imposed possibly by differences in environments from rainfall, seasonality, human and vector hosts as well as varied levels of interventions. One of the countries which remain heavily affected is Nigeria. Nigeria bears the highest burden of malaria (mostly from *Plasmodium falciparum* infections) globally and interventions with drugs and anti-vector tools suffer from logistic difficulties associated with the economy and size of the country as well as extensive infection movements [1]. This has been compounded by antimalarial drug resistance, evasion of host immune response and lack of highly efficacious vaccines [2–4]. It has been shown that high drug pressure and immunity select for adaptive parasite strains that sustain transmission (2, 5). Thus, several *P. falciparum* genes encoding drug or immune targets are under natural selection and bear signatures of directional or balancing selection [5–7]. For a more effective management of interventions, it is important to determine patterns of variation due to adaptation of the parasite to interventions and host environments. While directional forces cause adaptively important genetic variants of the parasite to increase in frequency leading to high fixation rates and the appearance of selective sweeps around affected loci, balancing selection brings the parasite’s favoured alleles to an intermediate equilibrium, where they are maintained as genetic polymorphisms. Although population genomics of *P. falciparum* explored in other West African populations have shown strong directional selection around known drug resistance genes and signatures of balancing selection on multiple candidate vaccine antigens [8–12], inferences of local adaptive mechanisms require looking more representatively at individual populations such as Nigeria.

Recent advances in next-generation genomics have proved useful in individual sequencing of *P. falciparum* in order to identify genes with polymorphic site frequency spectra consistent with selection. However, such technologies are still infeasible in resource-poor settings where malaria is endemic and large data sets are required. High-throughput sequencing on samples pooled from different individuals is a strategy that has been adopted to characterize genetic variability in diploid *Drosophila* at a small fraction of cost to detect single nucleotide polymorphisms (SNPs) and their estimated frequencies with a variance comparable with individual sequencing [13]. To understand the genome-wide patterns of selection in Nigeria, we sequenced pooled isolates of *P. falciparum* and identified genes that are most variable and potentially under strong directional and balancing selection from antimalarial drug use and host immunity.

## Methods

### Sample collection

Participants 2 years and above seeking care for uncomplicated malaria at three health centres - Aramoko-Ekiti (AMK), Lekki (LEK) and Badagry (BDG) - in southwestern Nigeria were recruited during the malaria season between March and September, 2013 (Additional file 1: Figure S1), [samples were collected as part of the *Plasmodium* Diversity Network in Africa (PDNA) project]. All recruited malaria cases had a temperature of > 37.5 °C on presentation or history of fever in the previous 48 h, and a minimum of 5000 *P. falciparum* parasites/μl estimated by thick film examination. Up to 5 ml of venous blood was collected from each participant in sterile ethylene diamine tetracetic acid (EDTA)-coated vacutainer tubes after which participants were treated with artemether-lumefantrine according to Nigeria’s treatment policy [14]. Collected samples were transported in an ice-cold container to the UK Medical Research Council Laboratories, The Gambia for further analyses.

### Parasite examination and pooled sequencing

Thick and thin blood films were prepared and examined under the microscope (Olympus CX21, Hamburg, Germany) for confirmation of falciparum infections. *Plasmodium falciparum-*infected blood was depleted of white blood cells by size selection and hydrophoresis using CF11 columns to reduce the presence of human DNA [15]. Thick and thin blood films were prepared before and after cellulose filtration to confirm complete removal of leucocytes. Extraction of genomic DNA was carried out using Qiagen Mini Kit (Qiagen, Hilden, Germany). The extracted DNA samples were stored at -20 °C for subsequent analysis. The quality as well as quantity of each DNA sample was determined using nanodrop (Thermo Fisher Scientific, MA, USA). Real-time PCR was carried out to determine the quantity of parasite DNA relative to human DNA contained in each sample using the procedures described by Veron et al. [16]. To further deplete human DNA extracted with the parasite DNA, NEBNext Microbiome DNA Enrichment Kit (New England Biolabs, MA, USA) was used. Methylated human DNA was removed from the DNA mixture by binding to the methyl-CpG binding domain of human MBD2-Fc protein [17]. Concentrations of individual parasite samples were normalized to 500,000 parasites/μl in a final volume of 920 μl of pooled DNA. Enzymatic shearing, end-repair and adapter-ligation leading to library preparation were carried out using NEBNext library prep kit (New England Biolabs, MA, USA). Library DNA was loaded on Ion 318™ chip (Thermo Fisher Scientific, MA, USA) and run on the Ion Personal Genome Machine (Thermo Fisher Scientific, MA, USA).

### Data processing: alignment, variant calling and quality filtering

Reads generated were aligned to *P. falciparum* 3D7 genome using mapping and assembly with quality (MAQ) tools. We excluded reads of poor mapping quality, and derived a list of SNPs [and small insertions and deletions (indels)] based on uniquely mapping reads and acceptable levels of coverage (minimum 10, maximum 2000). In addition, we applied a filter to rule out error-prone variant calls, based on a threshold of Q20. Allele frequencies in the population were determined for all SNPs, by analyzing all genotyped samples. Non-reference (variant) allele frequency (NRAF) was computed as the proportion of genotyped samples whose allele was not the reference allele. The reading frame and exon boundaries were determined from the PlasmoDB 13.0 annotation of the 3D7 genome (www.plasmodb.org).

### Identifying genes under balancing and directional selections

The allele frequency-based neutrality test, Tajima’s D [18], integrated haplotype score [19], and the ratio of nonsynonymous and synonymous polymorphisms (π_N_/π_S_) were applied to identify genes under selection. DnaSP 5.1 [20] was used to perform calculation of Tajima’s D ratios. Evidence of recent directional selection was obtained from the standardised haplotype score using WHAMM [19]. Extreme positive iHS scores (> 2.5) suggested selective sweeps along the ancestral allele signaling strong directional selection around that site.

## Results

**Fig. 1:**
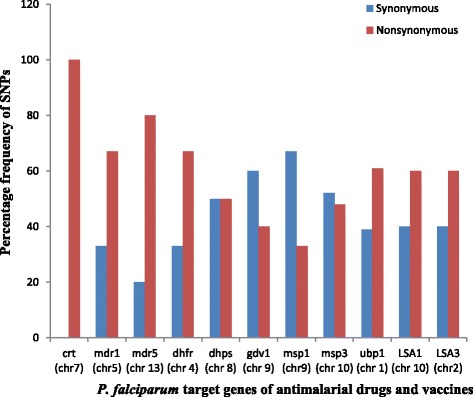
Distribution of synonymous and nonsynonymous SNPs among known gene targets of drug and immunity. *Abbreviations*: crt, chloroquine resistant transporter; mdr, multidrug resistant transporter; dhfr, dihydrofolate reductase; dhps, dihydropteroate synthase; gdv, gametocyte development; msp, merozoite surface protein; ubp1, deubiquitinating enzyme gene 1; lsa, liver stage antigen

High quality sequence data was obtained from genomic DNA pooled from 100 clinical isolates of *P. falciparum*. Genome-wide short-read sequences were generated yielding a median short sequence read length of 182 bp. Alignment to *P. falciparum* 3D7 reference genome indicated an average genome-wide coverage depth of 5.7× (Table 1). The sequencing technology yielded 488 million bases and total usable reads of 3,090,240. Variant calls were made at 56,784 polymorphic sites and 13,784 SNPs were identified after quality filtering. There was > 65% more read coverage in the genic regions than in the intergenic regions. Five thousand one hundred and twenty-two genes had at least 3 SNPs and were considered informative for frequency-based analyses. In all, 3122 genes were analysed across the 14 chromosomes of the parasite after excluding genes in the sub-telomeric regions (var, rifin and stevor genes). Each SNP was classified as synonymous or nonsynonymous according to whether an amino acid change occurred when substituting the reference allele with the non-reference allele at that SNP in the 3D7 reference genome sequence, without any other changes. Comparison between nonsynonymous and synonymous SNPs for some known target genes of antimalarial drugs and immunity is presented in Fig. 1.

**Table 1:** Summary of sequence mapping to the reference *P. falciparum* genome

Alignment summary	Mapping characteristics
Length of non-gap regions covered by reads	13,226,305
Length of 24 bp unique regions of the reference	264,683
Reference nucleotide composition	A: 37.87%, C: 13.35%, G: 14.62%, T: 34.16%
Reads nucleotide composition	A: 36.07%, C: 15.71%, G: 17.71%, T: 30.51%
Average depth across all non-gap regions	0.691
Average depth across 24 bp unique regions	0.514
Number of reference sequences	132,994
Length of reference sequences excluding gaps	53,277,940
Length of gaps in the reference sequences	1,299,037
Average coverage depth of reference sequence	5.7×

### Evidence of positive directional selection

Standardised integrated haplotype score (iHS) was used to examine evidence of recent directional selection. Fourteen shared iHS regions that had at least 2 SNPs with a score > 2.5 were identified (Table 2). Two of such genes were chloroquine resistance transporter (CRT) on chromosome 7 and multidrug resistance 1 (MDR1) on chromosome 5. There was a weak signature of selection in dihydrofolate reductase (DHFR) in chromosome 4 and MDR5 genes on chromosome 13 with only 2 and 3 SNPs respectively identified within the iHS window (Table 3). However, there was no evidence of recent directional selection in dihydropteroate synthase (DHPS) gene on chromosome 8. There was also a major selective sweep on chromosome 6 which had 32 SNPs within the shared iHS region. However, the specific loci targeted by the selective sweep were unknown.

**Table 2:** Windows of directional selection for genomic regions with shared iHS score 2.5

Chromosome	Window		Region (kb)	Number of SNPs with iHS > 2.5	ID of genes within region
	Start	Stop			
1	163	184	21	4	PF3D7_0103600 - PF3D7_0104200
2	302	515	213	1	PF3D7_0210900 - PF3D7_0213600
4	407	585	178	2	PF3D7_0409600 - PF3D7_0413300
5	822	1007	185	12	PF3D7_0519800 - PF3D7_0524200
6^a^	1039	1297	258	32	PF3D7_0625100 - PF3D7_0630600
7	238	249	11	4	PF3D7_0704550 - PF3D7_0704900
9	1060	1220	160	15	PF3D7_0929320 - PF3D7_0930500
10	1210	1557	347	10	PF3D7_1029500 - PF3D7_103880
11	1190	1310	200	8	PF3D7_1132920 - PF3D7_1133600
12	1451	1880	429	6	PF3D7_1234900 - PF3D7_1248500
13	303	358	55	3	PF3D7_1306470 –PF33D7_130790
13	2410	2520	110	3	PF3D7_1306470 - PF3D7_1307900
14	1910	1932	22	2	PF37_1407000 - PF3D7_1447900

^a^Selective sweep on chromosome 6 with 32 SNPs within the shared iHS region

**Table 3:** Distribution of SNPs in some target genes of antimalarial drugs and immunity

Gene Name	Chromosome	Position	Number of SNPs
UBP1	1	190,269–210,230	4
LSA3	2	796,752–801,568	6
PFDHR	4	748,088–749,914	13
PFMDR1	5	957,890–962,149	8
PFCRT	7	403,222–406,317	5
PFDHPS	8	548,200–550,616	7
MSP1	9	1,201,812–1,206,974	6
GDV1	9	1,377,953–1,379,752	4
MSP3	10	1,404,195–1,405,259	5
LSA1	10	1,436,316–1,439,804	5
PFMDR5	13	1,598,401–1,601,178	10

*Abbreviations*: *UBP1* ubiquitinating emzyme gene, *CRT* chloroquine resistant transporter, *MDR* multidrug resistant transporter, *DHFR* dihydrofolate reductase, *DHPS* dihydropteroate synthase, *GDV* gametocyte development, *MSP* merozoite surface protein, *LSA* liver stage antigen

### Identifying signatures of balancing selection

**Fig. 2:**
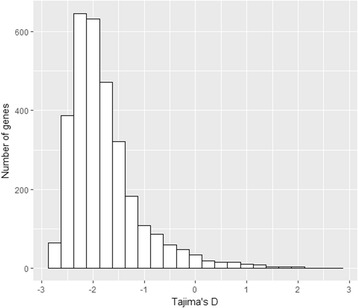
Frequency distribution of Tajima’s D values for genic loci as annotated in *Plasmodium falciparum* 3D7 v3 genome

Tajima’s D values were mostly negative (Additional file 2: Table S1), with a mean value of -0.86. One hundred and twelve genes (3.59%) had positive Tajima’s D values (Fig. 2). Six genes that have been previously associated with selection from acquired immunity had significantly high Tajima’s D values (Table 4).

**Table 4:** Genes showing high values of Tajima’s D

Gene ID	Chromosome number	Frequency of polymorphism π × 10^-3^	Tajima’s D
MSPDBL2	10	27.9	2.58
CSP	3	20.6	1.38
EBA-175	7	17.2	1.29
MSP7	13	13.5	0.84
MSP3	10	23.8	0.73
SERA5	2	24.1	0.21

*Abbreviations*: *MSPDBL2* merozoite surface protein duffy binding-like, *CSP* circumsporozoite protein, *EBA-175* erythrocyte-binding antigen, *MSP* merozoite Surface Protein, *EBA* erythrocyte-binding antigen, *SERA* serine repeat antigen

## Discussion

This investigation adopted pooled DNA sequencing to understand genome-wide patterns of selection and genetic variability that had only been previously achievable through individual sequencing [8, 12]. Regions of the genome under recent positive and balancing selections, representing signatures of adaptation to drug pressure and host immunity, were identified. Earlier reports showed no significant variance or population sub-structuring in the study areas [21], eliminating the possibility of false-positive results for signals of selection owing to population structure.

Drug pressure is a powerful selective force in natural parasite populations. Removal of drug pressure exposes resistant parasites to increased competition leading to a decline in the frequency of resistance conferring selection [22], as reported in Malawi where after just a decade of non-use, chloroquine (CQ) cleared 100% of asymptomatic *P. falciparum* infections [23]. Similarly, the prevalence of CQ-resistant parasites in coastal Tanzania decreased after only two and a half years of CQ withdrawal [24]. However, in keeping with the observation of high distribution of CQ-resistant parasites in western Kenya after a period of drug withdrawal [25], the results of the present study showed that the population of resistant parasites in the Nigerian region studied did not reduce significantly after years of CQ withdrawal. Instead, high extended haplotype scores (iHS) were found for SNPs around chloroquine resistant transporter (CRT) and multidrug resistance (MDR1) genes. These observations are consistent with positive selection from long periods of chloroquine use leading to depletion of chloroquine-susceptible parasites [14]. Strong directional selection in these genes essentially suggests that the re-introduction of the drug for malaria treatment cannot be considered yet as drug resistant strains still predominate the population. This is contrary to previous observations which showed complete [23] or partial [24] reversal of *P. falciparum* populations to chloroquine susceptibility after a period of drug withdrawal. In populations where malaria is endemic, transmission is high, and acquired immunity is extensive, asymptomatic adults, who rarely become ill and who generally do not receive therapy, may provide a favorable environment for susceptible parasites to persist in the population. Malaria intervention efforts in Nigeria might have resulted in lower transmission and less acquired immunity, shrinking refugia and allowing resistant alleles to become fully fixed in the population, as was observed in South America [26] and Southeast Asia [27].

There was no strong evidence of selection associated with dihydrofolate reductase (DHFR) and dihydropteroate synthase (DHPS), the two major genes targeted by sulfadoxine-pyrimethamine (sulfadox). This supports reports that the drug is still considerably effective for malaria treatment [28]. Thus, its use as Intermittent Preventive Treatment (IPT) of pregnant women remains effective. However, the detection of weak selection on DHFR gene implies selection acting in the drug resistance locus as sulfadox is still widely available from the informal pharmaceutical sector. There was also a major selective sweep on chromosome 6 supporting previous reports from Senegal [29] and The Gambia [9]. This is the first evidence of the Chromsome 6 sweep beyond the populations of Senegal and The Gambia which are geographically linked. It therefore could be marking a common selective event in the history of *P. falciparum* in West Africa. However, further investigation is needed to understand the origin and mechanisms of selection in the chromosome region.

Against a genomic background in which most genes had negative values of Tajima’s D, this study, in agreement with observations in Senegal [12] and The Gambia [8], has identified six genes to be under strong balancing selection. These are some of the antigens that had been previously proposed as vaccine candidates [8, 9]. However, not all genes with high positive values of Tajima’s D are under the effect of balancing selection as demographic events such population contraction could also lead to values greater than zero. Thus, the parasite surface genes identified to be under balancing selection may require further validation for selection from immunity or diversity of human erythrocyte receptors. Immunological analyses of the allelic protein products of these identified genes should be prioritized.

While pool-seq has been shown here to be a useful approach in population genomics of *P. falciparum*, it is worthy of note that haplotype information may be traded off under multiplexed sequencing. Although this may be a necessary price for a cost-effective and accurate characterization of SNP frequencies, it should not be considered insignificant. Also, loss of resolution on haplotype structure makes it difficult to separate demographic from complete selective events as patterns of linkage disequilibrium (LD) cannot be resolved [12]. Futher application of this approach on *P. falciparum* evolutionary analyses will benefit from a more detailed comparison of allele frequency spectrum obtained with that from individual sequencing.

## Conclusion

We have demonstrated the use of pooled DNA sequencing to understand genome-wide patterns of selection of *P. falciparum* previously achievable through individual sequencing, identifying regions of the parasite genome under the influence of drug and host immune selection.

## Additional files

Additional file 1: Figure S1. Map of the study area in South Western Nigeria. Shaded areas indicate regions of study and lines linking regions show the spatial distance (km) between sites. (PDF 89 kb)

Additional file 2: Table S1. Tajima’s D values for known genes. (XLSX 89 kb)

## Abbreviations

CRT: Chloroquine resistance transporter
DHFR: Dihydrofolate reductase
DHPS: Dihydropteroate synthase
EBA: Erythrocyte binding antigen
iHS: Integrated haplotype score
MAQ: Mapping and assembly with quality
MDR: Multidrug resistance
MSP: Merozoite surface protein
NRAF: Non-reference allele frequency
SERA: Serine repeat antigen
sSA: Sub-Saharan Africa
