# Supplementary figures and images for: Pooled-DNA sequencing identifies genomic regions of selection in Nigerian isolates of Plasmodium falciparum
Source: Parasit Vectors. 2017 Jun 29;10:320. doi: 10.1186/s13071-017-2260-z (PMC5492182; doi:10.1186/s13071-017-2260-z)

Figure S1

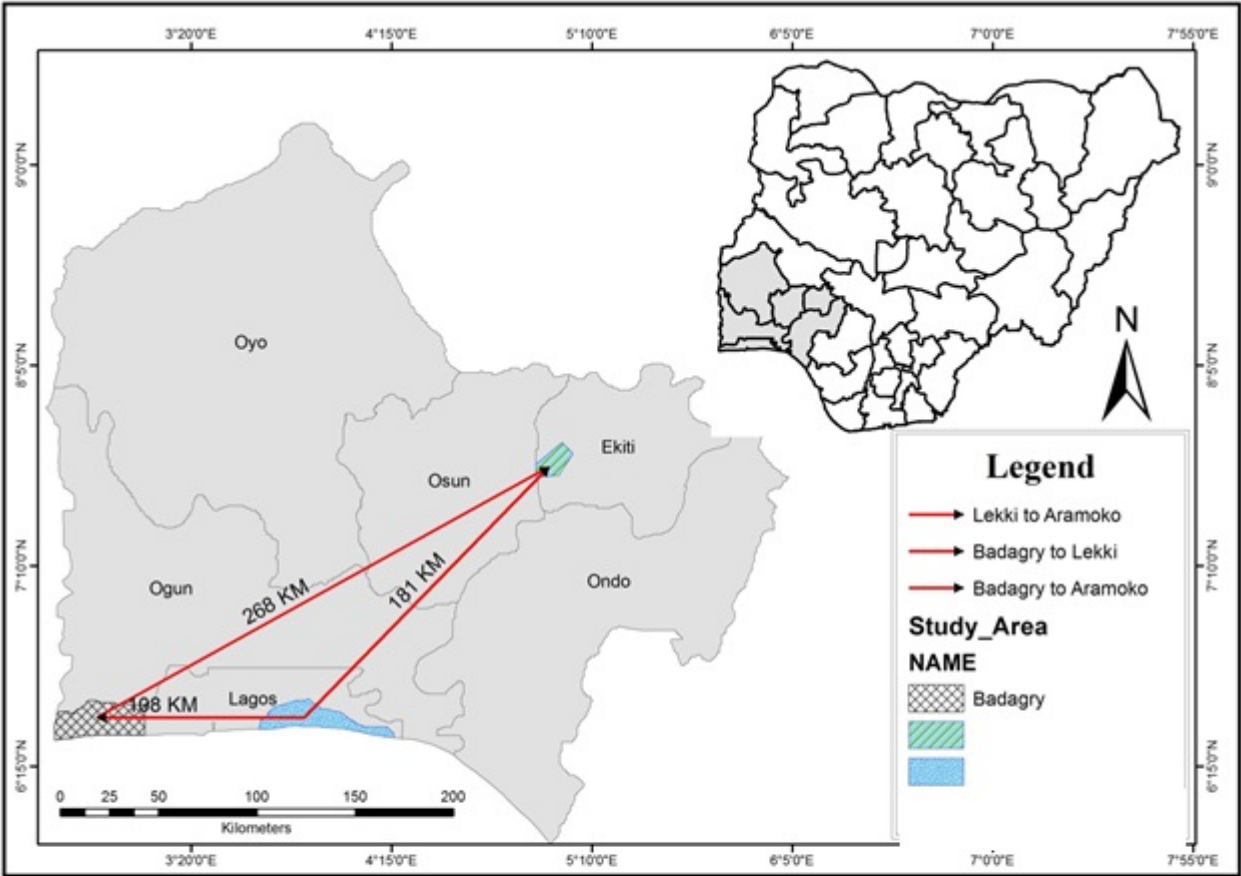

Supplement: Supplementary file 1 — Map of the study area in South Western Nigeria. Shaded areas indicate regions of study and lines linking regions show the spatial distance (km) between sites. (PDF 89 kb) [file 13071_2017_2260_MOESM1_ESM.pdf]
